# L-Citrulline Protects Skeletal Muscle Cells from Cachectic Stimuli through an *iNOS*-Dependent Mechanism

**DOI:** 10.1371/journal.pone.0141572

**Published:** 2015-10-29

**Authors:** Daniel J. Ham, Benjamin G. Gleeson, Annabel Chee, Dale M. Baum, Marissa K. Caldow, Gordon S. Lynch, René Koopman

**Affiliations:** Basic and Clinical Myology Laboratory, Department of Physiology, The University of Melbourne, Parkville, Victoria, Australia; University of Canberra, AUSTRALIA

## Abstract

Dietary L-citrulline is thought to modulate muscle protein turnover by increasing L-arginine availability. To date, the direct effects of increased L-citrulline concentrations in muscle have been completely neglected. Therefore, we determined the role of L-citrulline in regulating cell size during catabolic conditions by depriving mature C2C12 myotubes of growth factors (serum free; SF) or growth factors and nutrients (HEPES buffered saline; HBS). Cells were treated with L-citrulline or equimolar concentrations of L-arginine (positive control) or L-alanine (negative control) and changes in cell size and protein turnover were assessed. In myotubes incubated in HBS or SF media, L-citrulline improved rates of protein synthesis (HBS: +63%, SF: +37%) and myotube diameter (HBS: +18%, SF: +29%). L-citrulline treatment substantially increased *iNOS* mRNA expression (SF: 350%, HBS: 750%). The general NOS inhibitor L-NAME and the *iNOS* specific inhibitor aminoguanidine prevented these effects in both models. Depriving myotubes in SF media of L-arginine or L-leucine, exacerbated wasting which was not attenuated by L-citrulline. The increased *iNOS* mRNA expression was temporally associated with increases in mRNA of the endogenous antioxidants *SOD1*, *SOD3* and *catalase*. Furthermore, L-citrulline prevented inflammation (LPS) and oxidative stress (H_2_O_2_) induced muscle cell wasting. In conclusion, we demonstrate a novel direct protective effect of L-citrulline on skeletal muscle cell size independent of L-arginine that is mediated through induction of the inducible NOS (*iNOS*) isoform. This discovery of a nutritional modulator of *iNOS* mRNA expression in skeletal muscle cells could have substantial implications for the treatment of muscle wasting conditions.

## Introduction

Skeletal muscle wasting, the loss or atrophy of skeletal muscle, is a serious complication in many diseases and conditions including chronic heart failure, sepsis and cancer [1]. The loss of muscle mass and function can impact on mobility and reduce quality of life, particularly in at-risk populations such as the elderly. As such, the development of strategies to prevent muscle wasting is of major importance.

Muscle wasting results from a chronic imbalance between rates of muscle protein synthesis and breakdown, with breakdown exceeding synthesis [2]. Protein metabolism is tightly regulated by nutrient availability, especially amino acids. Since the discovery that essential amino acids, particularly leucine, stimulate muscle protein synthesis [3], the regulation of muscle protein metabolism by non-essential amino acids has been largely ignored. Interestingly, over the last decade the non-proteogenic amino acid L-citrulline has been touted as a potential nutritional intervention for muscle wasting. This hypothesis stems from the observation that ingestion of L-citrulline increases blood and muscle concentrations of L-arginine more than oral L-arginine [4–8]. In contrast to L-arginine, L-citrulline is not metabolized in the gut or taken up by the liver and the majority (~75%) of oral L-citrulline is converted to L-arginine in the kidney [9, 10]. As such, L-citrulline supplementation has proved effective at restoring muscle L-arginine stores and reducing muscle wasting in L-arginine-deficient and low-protein intake conditions [6, 11, 12]. L-arginine is important because it provides substrate for the production of creatine and proteins, and because it is the primary substrate for nitric oxide (NO) synthesis [13]. Therefore, sufficient L-arginine availability is crucial for the maintenance of skeletal muscle size both *in vitro* [14] and *in vivo* [6, 11, 12]. Dietary intake of L-citrulline also markedly increases the plasma availability of L-citrulline [5, 8], but the effects of increased L-citrulline availability on skeletal muscle cells subjected to cachectic stimuli have not been studied.

Within some cell types (e.g. endothelial and neuronal cells) L-citrulline is intimately involved in the re-supply of L-arginine for the synthesis of NO by nitric oxide synthase (NOS) [15, 16]. NOS catalyzes the production of NO and L-citrulline from L-arginine. Subsequently, L-citrulline can be recycled to L-arginine by the enzymes argininosuccinate synthase (ASS1) and argininosuccinate lyase (ASL). Therefore, in cell types that rely heavily on NO as a signaling molecule (e.g. relaxation of smooth muscle cells), L-citrulline serves as an L-arginine precursor and the effects of exogenous L-citrulline are analogous with L-arginine [16]. The role of NO in skeletal muscle homeostasis is diverse, playing an important signaling role in satellite cell activation [17], myoblast fusion [18], regeneration [19] and overload-induced skeletal muscle hypertrophy [20, 21]. In addition, NO production may serve as a protective mechanism against catabolic stimuli [22]. As L-arginine is the key substrate for the production of NO it is tempting to assume that L-arginine mainly effects muscle in a NO-dependent manner. However, although exogenous L-arginine directly modulates muscle protein metabolism and attenuates muscle wasting in C2C12 myotubes, we recently demonstrated that the effect of L-arginine was not dependent on the production of NO [14]. In contrast, L-arginine exerts its effect through the more classical amino acid sensitive mTORC1 signaling pathway. To date, the potential role of L-citrulline in regulating skeletal muscle protein metabolism and cell size remains to be established.

In this study, we investigated the direct effects of L-citrulline on skeletal muscle cells and identified a novel protective effect of L-citrulline on protein metabolism and skeletal muscle cell size independent of L-arginine. We show these effects are mediated through the inducible NOS (iNOS) isoform and describe for the first time, a nutritional modulator of iNOS expression in skeletal muscle cells that could have important implications for muscle wasting conditions.

## Materials and Methods

### Cell culture

**Fig 1:**
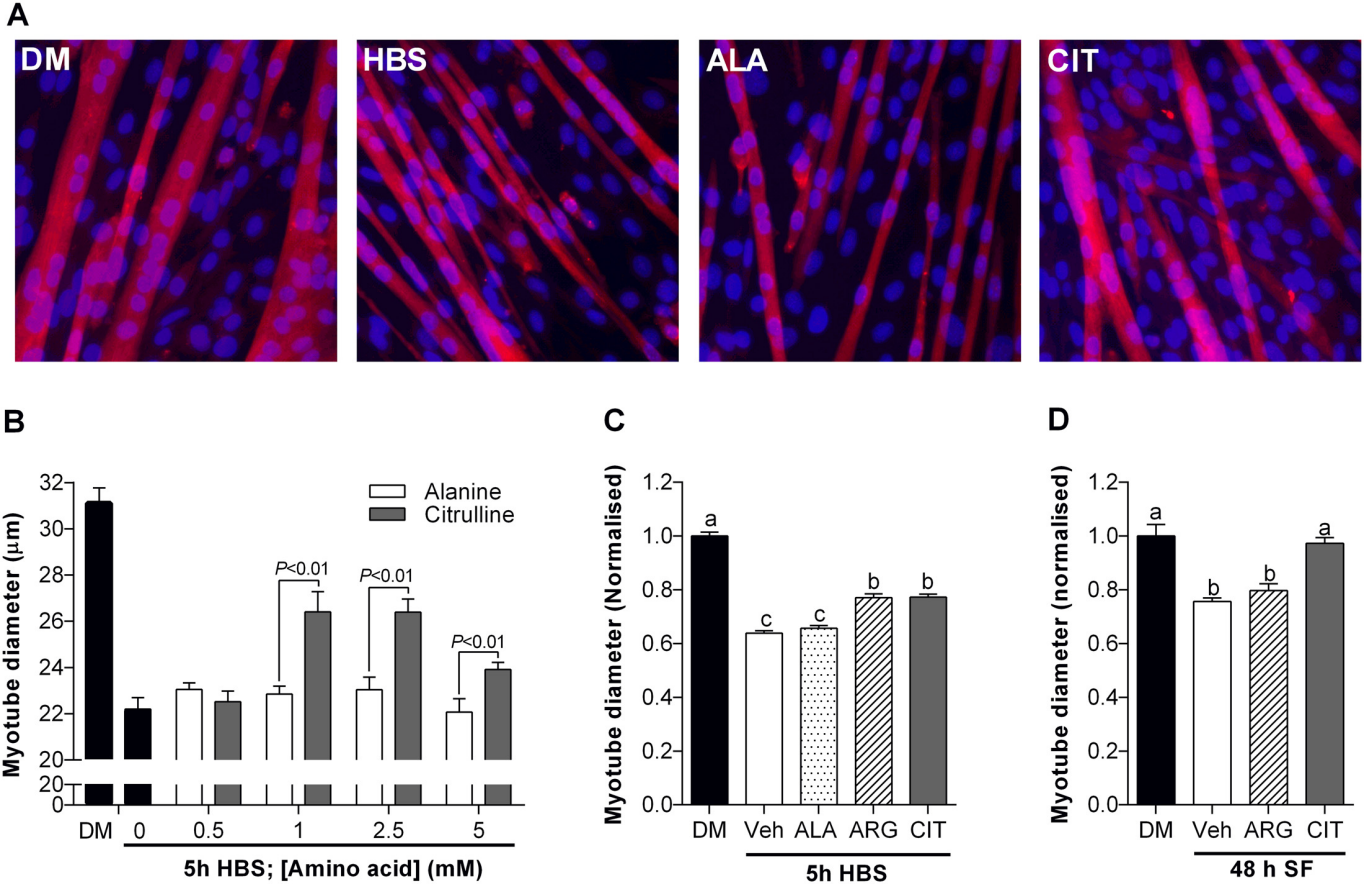
L-citrulline protects C2C12 muscle myotubes from wasting. Myotube diameter after 5 h incubation in HEPES buffered saline (HBS) with increasing concentrations of L-citrulline or isomolar concentrations of L-alanine (B) and representative images (20× objective) at the optimal dose of 2.5 mM (A). Myotube diameter for cells incubated in differentiation media (DM) or: HBS for 5 h (C) or; serum free (SF) media for 48 h (D). In each model, cells were treated with vehicle (PBS), 2.5 mM L-arginine or 2.5 mM L-citrulline (n = 5–8 per group). Values are means ± SE. For (B) comparisons were made using a two-way ANOVA (treatment × dose) with Fisher’s LSD post-hoc test. Significant differences are displayed where appropriate. For (C-D), Comparisons were made using a one-way ANOVA with Tukey’s post hoc test. Different letters denote significant differences (*P*<0.05) between groups, where a>b>c.

Murine C2C12 myoblasts (Cryosite distribution, NSW, Australia) were plated in 6 or 12 well plates and cultured in DMEM (Life Technologies, Australia) containing 10% (v/v) fetal calf serum (Life Technologies) and antimycotic antibiotic solution (100 unit/ml penicillin/streptomycin, Life Technologies) at 37°C in an atmosphere of 5% CO_2_. Upon confluency, the media was changed to DMEM containing 2% (v/v) horse serum (Life Technologies) for 5 days to promote formation of mature multinucleated myotubes [14]. To induce wasting, cells were: 1) washed once in HEPES buffered saline (HBS) and then incubated in HBS for 5 h, as previously described [14]; 2) washed once in serum free DMEM (Life Technologies, Australia) and then incubated in serum free DMEM for 48 h (SF); 3) incubated in DMEM containing 1 μg.ml^-1^ LPS for 24 h; or 4) incubated in DMEM containing 25 μM H_2_O_2_ for 24 h. L-arginine, leucine and lysine free DMEM was purchased from Life Technologies (Australia) and appropriate concentrations of lysine, and L-arginine or leucine were added back into the media to obtain L-arginine-free DMEM and leucine-free DMEM. All amino acids were purchased from Sigma-Aldrich (Castle Hill, NSW, Australia). Based on an initial dose-ranging experiment (Fig 1), 2.5 mM of L-citrulline or control amino acids (L-alanine and L-arginine) was chosen for all future experiments.

We determined in preliminary experiments that iNOS protein was below detectable limits in all of our samples using standard western blotting (data not shown). This is in line with previous research, where measurable changes in iNOS protein are only observed under pro-inflammatory conditions (e.g. LPS) [22]. Furthermore, we did not detect a change in total nitrate/nitrite in the media of citrulline treated cells using a commercial fluorometric assay (Cayman Chemical Company, Ann Arbor, MI, USA; data not shown). As it is likely that the citrulline-induced changes in iNOS activity are highly localised within the cells, and therefore difficult to detect, we chose to use ‘loss of function’ inhibitor studies to demonstrate the requirement of NOS activity for the protective effect of citrulline. To this end, L-NG-NitroL-arginine Methyl Ester (L-NAME, 10 mM, Sigma-Aldrich) was used to inhibit NO production by all NOS isoforms [18] and aminoguanidine (0.5 mM, Sigma-Aldrich) was used as an iNOS specific inhibitor [23]. Rapamycin (100 nM, Sigma-Aldrich) was used to inhibit mTORC1 activation [24]. A 30 min pre-treatment period was used for all inhibitors. Sodium nitroprusside (SNP, 0.2 mM, Sigma-Aldrich) was used as a NO donor [25].

### Determination of myotube diameter

Cells were grown in 12-well plates and prepared for immunohistochemistry as described previously [14]. Briefly, cells were washed in phosphate buffered saline (PBS), fixed with 3.7% formaldehyde for 15 min, permeabilized with 0.3% TritonX100 for 5 min and then incubated in anti-α-myosin (1:50, Sigma-Aldrich) in PBS at room temperature for 1 h. Cells were then washed in PBS and incubated in goat-anti-rabbit Alexa555 secondary antibody (1:400, Life Technologies) and DAPI (1:1000) for 30 min in PBS. Cells were washed in PBS and then imaged on a Zeiss Axiovert 40 CFL inverted microscope at 20× magnification. Four images were taken in each well from pre-defined locations within each quadrant. Myotube diameter was measured using Axiovision 4.8 software (Zeiss, USA). A total of ~50–80 myotubes were measured per well and the average diameter of each well was used for statistical analysis. To confirm myotube viability, myotubes incubated in DM or SF meida for 48 h were incubated in 0.4% Trypan Blue in PBS for 2 min, washed, fixed and imaged as described. Following our standard myotube imaging protocol, no Trypan Blue positive myotubes were observed in any treatment group (S1 Fig). However, when searched for specifically, a few Trypan Blue positive cells could be seen in each treatment group. The number of viable cells in each treatment group are likely to be >99.0–99.5%.

### Determination of protein synthesis

Myotubes were grown in 6-well plates and treated as described (cell culture). To determine the rate of protein synthesis we utilized SUnSET methodology, as described [14, 26]. Briefly, puromycin (Sigma-Aldrich) was administered to the media at a final concentration of 1 μM exactly 30 min before cells were collected in ice-cold homogenizing buffer as described [14]. Anti-puromycin was purchased from Merck Millipore (Kilsyth, Victoria, Australia).

### Protein extraction and western blotting

Cell lysates were homogenized in ice-cold extraction buffer and centrifuged at 10000 g for 10 min at 4°C to remove cell debris as previously described [14]. Briefly, supernatant protein concentrations were determined using the Bradford-Lowry protein assay method, as per the manufacturer’s instructions (Bio-Rad Laboratories, NSW, Australia) and prepared for immunoblotting. Protein (30 μg/lane) was separated by SDS-PAGE gels and proteins were transferred to 0.45 mm PVDF via Trans-Blot^®^ Turbo™ transfer system (Bio-Rad Laboratories). Membranes were blocked for 1 h at room temperature (RT) in 5% (w/v) bovine serum albumin (BSA, Sigma-Aldrich) in Tris-buffered saline-Tween 20 (TBST) then incubated overnight at 4°C with primary antibodies (pmTOR (S2448), mTOR, p4EBP1 (T37/46) and 4EBP1; Cell Signaling Technologies, Beverly, MA, USA) diluted 1:1000 in 5% BSA/TBST. The following day membranes were washed (5 × 5 min in TBST) and then incubated for 1 h at RT in HRP-conjugated secondary antibodies (mouse anti-rabbit or goat anti-mouse immunoglobulins; GE Healthcare Life Sciences, Australia) diluted in 5% BSA/TBST. After washing (5 × 5 min in TBST), proteins were visualized by enhanced chemiluminescence (Super Signal West Femto; Thermo Scientific) using the ChemiDoc™ imaging system (Bio-Rad Laboratories) and quantified using ImageLab 4.0 software (Bio-Rad Laboratories). Proteins of interest were normalized to total protein as determined by BLOT-FastStain™ as per manufacturer’s instructions (G-Biosciences, St Louis, MO).

### RNA extraction and qPCR

Cells were lysed in RLT buffer (Qiagen, VIC, Australia) and total RNA extracted according to the manufacturer’s instructions (RNeasy Mini Kit; Qiagen). Briefly, RNA quality and concentration were determined using the Nanodrop 1000 (Thermo-Fisher Scientific, Australia). First-strand cDNA was generated using 100 ng of total RNA using the SuperScript™VILO cDNA Synthesis Kit according to manufacturer’s instructions (Life Technologies, VIC, Australia). qPCR was performed in duplicate using the Bio-Rad CFX384 PCR system (Bio-Rad Laboratories) with reaction volumes of 10 μl, containing SsoAdvanced™ Universal SYBR^®^ Green Supermix (Bio-Rad Laboratories), forward and reverse primers and cDNA template (2 ng/μl). Gene expression was quantified by normalizing raw Cq values to the cDNA content of each sample and expressed as arbitrary units (AU). Primers were designed using NCBI primer BLAST and specificity confirmed using Basic Local Alignment Search Tool (BLAST) (primers are listed in Table 1). A melting point dissociation curve was generated by the PCR instrument for all PCR products to confirm the presence of a single amplified product.

**Table 1:** Details of primers used for qRT-PCR analysis. Primer sequences were designed using NCBI primer BLAST using sequences accessed through GenBank and checked for specificity using nucleotide-nucleotide BLAST search.

Gene	GenBank Accession Number	Forward Primer (5’-3’)	Reverse Primer (5’-3’)
*Catalase*	NM_009804	ACCAAGGTTTGGCCTCACAA	TCCGGAGTGGGAGAATCCAT
*eNOS*	NM_008713	TGACCAGCACATTTGGCAATGG	CATGAGCGCTGCTGCAAAGC
*iNOS*	NM_010927	CACCTTGGAGTTCACCCAGT	ACCACTCGTACTTGGGATGC
*nNOS*	NM_008712	TTTCTGTCCGTCTCTCTTCAAACGCAAAGT	GCGGGAGACTGTTCGTTCTCTGAATACGGG
*SOD1*	NM_011434	GGAACCATCCACTTCGAGCA	CCCATGCTGGCCTTCAGTTA
*SOD2*	NM_013671	GCCCAAACCTATCGTGTCCA	AGGGAACCCCTAAATGCTGCC
*SOD3*	NM_ 011435	TTCTACGGCTTGCTACTGGC	GCTAGGTCGAAGCTGGACTC

### Statistical analyses

All values are expressed as mean ± SEM. Phosphorylated proteins were normalized to total protein of the protein of interest (p/t), while all other proteins were normalized to total protein for the whole lane (BLOT-FastStain™). All data were then normalized to the appropriate control group for ease of visualization. Data were tested for normality and homogeneity of variance using a Shapiro-Wilk and Levene’s test, respectively. For dose-response and time-course experiments, two-way ANOVAs (time/dose, treatment) with Fisher’s LSD post-hoc test were used for comparisons between groups, while one-way ANOVAs with Tukey’s post-hoc test were used for all other comparisons. *P*<0.05 was considered significant. Unless otherwise stated, data were normalized to control values.

## Results

### L-citrulline reduces muscle cell wasting in a dose-dependent manner in C2C12 myotubes

To determine the effect of L-citrulline on skeletal muscle cell size we first performed dose-response experiments while cells were deprived of growth factors and nutrients (HBS). The addition of L-citrulline to the HBS solution attenuated wasting in a dose-dependent manner, reaching statistical significance at a concentration of 1 mM (Fig 1). As compared to isomolar concentrations of L-alanine, myotube diameter was significantly greater (*P*<0.01) in cells treated with 1 mM (15.6%), 2.5 mM (14.5%) and 5 mM (8.4%) of L-citrulline (Fig 1). All further experiments were performed using 2.5 mM of L-citrulline and positive (L-arginine) and negative (L-alanine) control amino acids. The effect of L-citrulline on C2C12 myotube diameter was also explored using other models of muscle cell wasting and compared with the effects of isomolar amounts of L-arginine or vehicle (Fig 1). L-arginine and L-citrulline provided equal protection from wasting when cells were incubated in HBS for 5 h (*P*<0.01). In contrast, myotube diameter was significantly larger (*P*<0.01) in L-citrulline treated cells than vehicle or L-arginine treated cells incubated in serum free (SF) media for 48 h (Fig 1).

### L-citrulline protects muscle cells through a preservation of protein synthesis

**Fig 2:**
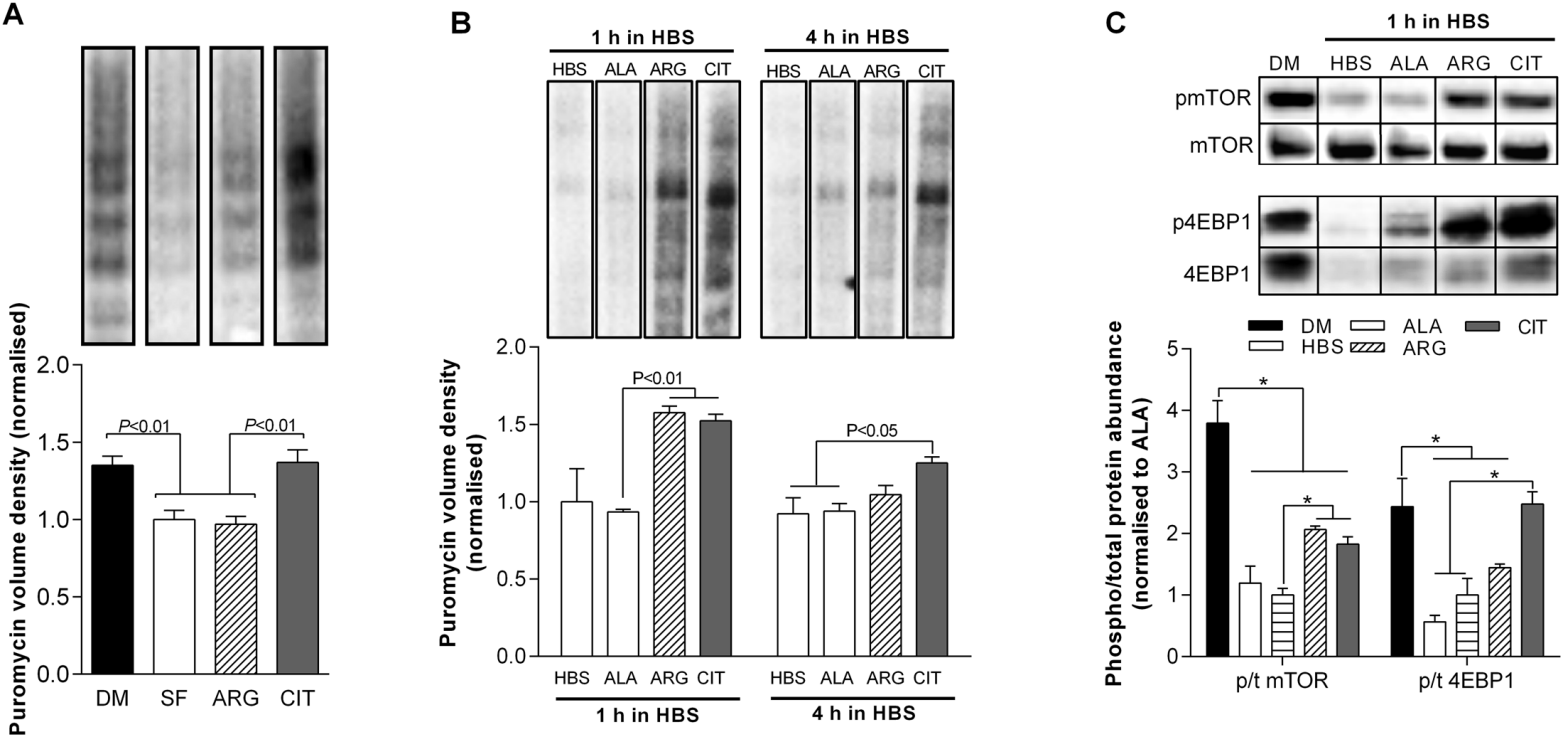
L-citrulline protects muscle cells through a preservation of protein synthesis. Protein synthesis as assessed using puromycin after 48 h in serum free (SF) media (A); and after incubation in HEPES buffered saline (HBS) for 1 and 4 h (B). Phosphorylation status (phospho/total protein) for mTOR and 4EBP1 after incubation in HBS for 1 h. Cells were treated with vehicle, 2.5 mM L-alanine (ALA), 2.5 mM L-arginine (ARG) or 2.5 mM L-citrulline (CIT). Values are means ± SE, n = 4–6 per group. Comparisons were made using a one-way ANOVA with Tukey’s post hoc test. Significant differences are indicated where appropriate. For each protein, all blots were performed on the same membrane.

After 48 h incubation in SF media, we observed a 26% reduction in protein synthesis, which was restored to CON levels with L-citrulline treatment (Fig 2A). Similarly, compared to L-alanine, protein synthesis was 63% and 33% higher in L-citrulline treated cells after 1 and 4 h incubation in HBS, respectively (P<0.01, Fig 2B). The phosphorylation status of mTOR and 4EBP1 were 74% and 59% lower in L-alanine treated cells after 1 h incubation in HBS than CON cells (P<0.01, Fig 2C). L-citrulline increased the phosphorylation status of mTOR by 83% (P<0.05) compared to L-alanine treated cells, but was still markedly lower than CON cells (P<0.01). In contrast, L-citrulline restored 4EBP1 phosphorylation to CON levels.

### L-citrulline requires the presence of essential amino acids to prevent muscle cell wasting

**Fig 3:**
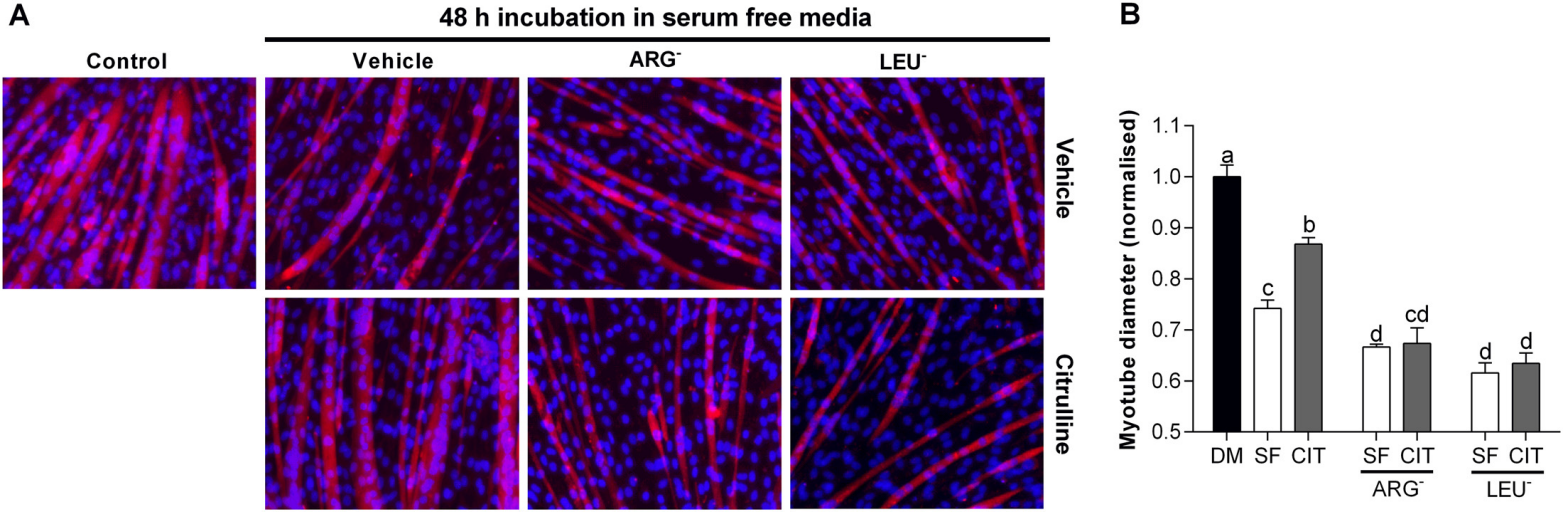
L-citrulline requires the presence of essential amino acids to prevent muscle wasting. Representative images (20× objective) (A) and myotube diameter (E) after 48 h incubation in serum free media (SF), L-arginine free (ARG^-^) and leucine free (LEU^-^) SF media. Comparisons were made using a one-way ANOVA with Tukey’s post hoc test. Different letters denote significant differences (*P*<0.05) between groups, where a>b>c>d and cd is not different to c or d.

Incubation of myotubes in L-arginine (ARG^-^) or leucine (LEU^-^) free media exacerbated SF-induced muscle cell wasting. The myotube diameter of cells incubated in ARG^-^ and LEU^-^ SF media were 10% and 17% smaller (*P*<0.05), respectively, than in SF media alone (Fig 3). L-citrulline attenuated SF-induced muscle cell wasting, but did not reduce muscle cell wasting under ARG^-^ or LEU^-^ conditions.

### L-citrulline increases the mRNA expression of *iNOS* and exogenous NO prevents SF-induced myotube wasting

**Fig 4:**
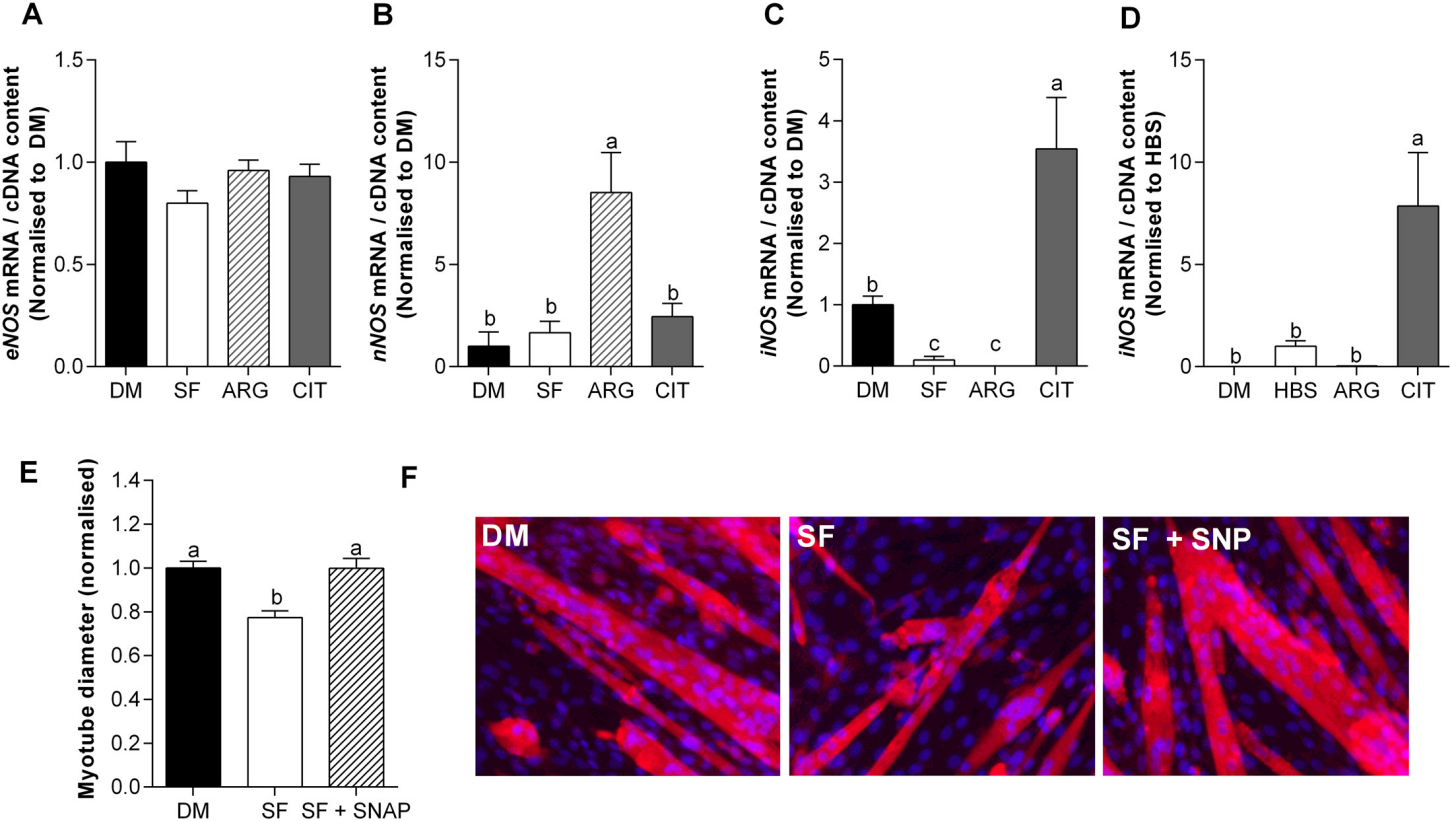
L-citrulline increases the mRNA expression of *iNOS* and exogenous NO prevents SF-induced muscle wasting. mRNA expression of *eNOS* (A); *nNOS* (B) and; *iNOS* (C) for DM and after 24 h in serum free (SF) media treated with vehicle, 2.5 mM L-arginine (ARG) or 2.5 mM L-citrulline (CIT). *iNOS* expression (D) after 5 h incubation in HEPES buffered saline (HBS). Myotube diameter (E) and representative images (20× objective) (F) after 48 h incubation in serum free media (SF). Cells were treated with vehicle (PBS) or 0.2 mM of the NO donor sodium nitroprusside (SNP). Values are means ± SE, n = 4–6 per group. Comparisons were made using a one-way ANOVA with Tukey’s post hoc test. Different letters denote significant differences (*P*<0.05) between groups, where a>b>c.

After incubating C2C12 myotubes in serum-free media for 24 h, the mRNA expression of *eNOS* and *nNOS* was unaltered, whereas *iNOS* was ~90% lower than in CON cells (P<0.01, Fig 3A–3C). While L-citrulline did not impact the mRNA expression of *eNOS* or *nNOS*, in CIT treated cells *iNOS* was ~35 fold higher than in SF (P<0.05), and ~3.5 fold higher than in CON treated cells (P<0.01), but, iNOS protein levels remained below detection limits (data not shown). We also observed an L-citrulline-induced increase in *iNOS* mRNA expression after 5 h of incubation in HBS (P<0.01, Fig 4D). To determine whether exogenous NO also conferred protection to myotubes, we treated cells in SF media with 0.2 mM of the nitric oxide donor SNP (Fig 4E and 4F). A dose of 0.2 mM SNP attenuated wasting such that SNP treated myotubes had similar diameters as the control cells. On the other hand, higher concentrations (1 mM) had deleterious effects (data not shown).

### *iNOS* activity is necessary for L-citrulline’s protective effect

**Fig 5:**
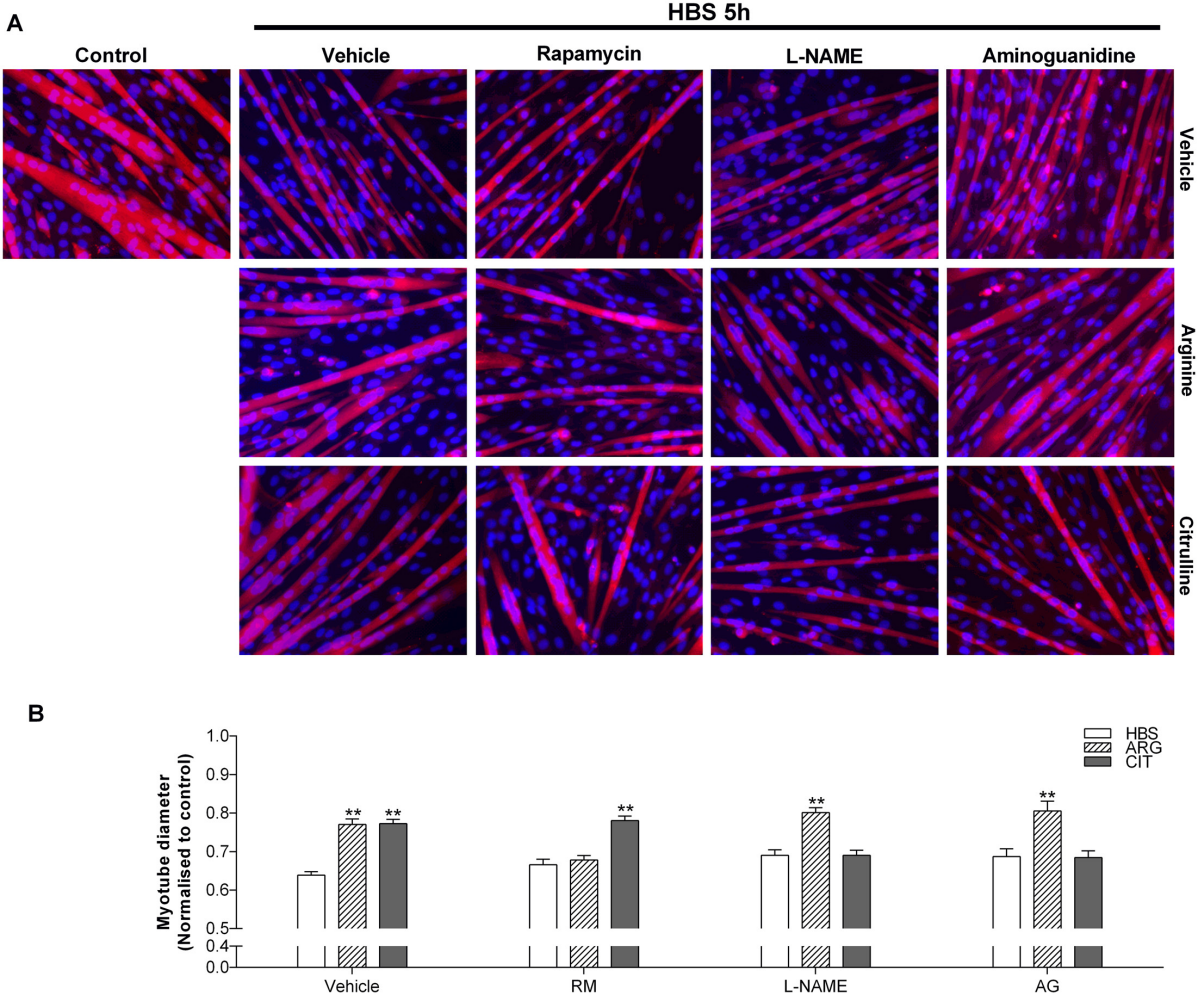
The protective effect of L-citrulline on HBS-induced myotube wasting is *iNOS*-dependent and mTORC1-independent. Representative images (20× objective) (A) and myotube diameter (B) after 5 h incubation in HEPES buffered saline (HBS). Cells were pre-treated with vehicle (PBS), rapamycin (RM), L-NAME or aminoguanidine (AG). 30 min later, cells were co-treated with vehicle (PBS), 2.5 mM L-arginine or 2.5 mM L-citrulline (n = 5–8 per group). Values are means ± SE. Comparisons were made using a one-way ANOVA with Tukey’s post hoc test. ** denotes a significant difference from vehicle at the P<0.01 level.

**Fig 6:**
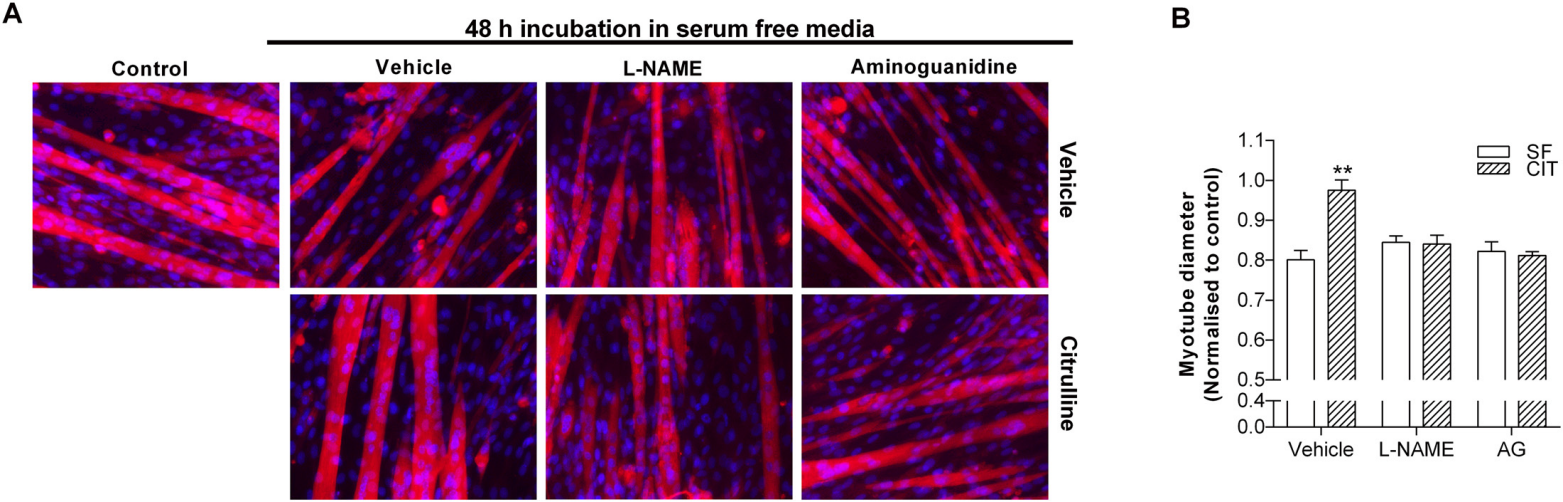
The protective effect of L-citrulline on SF-induced myotube wasting is *iNOS*-dependent. Representative images (20× objective) (A) and myotube diameter (B) after 48 h incubation in serum free media (SF). Cells were pre-treated with vehicle (PBS), L-NAME or aminoguanidine (AG). 30 min later, cells were co-treated with vehicle (PBS) or 2.5 mM L-citrulline (n = 5–8 per group). Values are means ± SE. Comparisons were made using a one-way ANOVA with Tukey’s post-hoc test. ** denotes a significant difference from vehicle at the P<0.01 level.

Both the general NOS inhibitor L-NAME [18] and the iNOS specific inhibitor aminoguanidine [23] completely prevented the protective effect of L-citrulline in both HBS (Fig 5) and SF (Fig 6) models of myotube wasting. In contrast, neither L-NAME nor aminoguanidine prevented the protective effect of L-arginine in HBS-induced muscle cell wasting (Fig 5). Interestingly, the mTORC1 inhibitor rapamycin prevented the protective effect of L-arginine but not L-citrulline on myotube diameter in the HBS model of muscle cell wasting (Fig 5), further highlighting the L-arginine-independent effects of L-citrulline.

### Increased *iNOS* expression is associated with increased antioxidant gene expression

**Fig 7:**
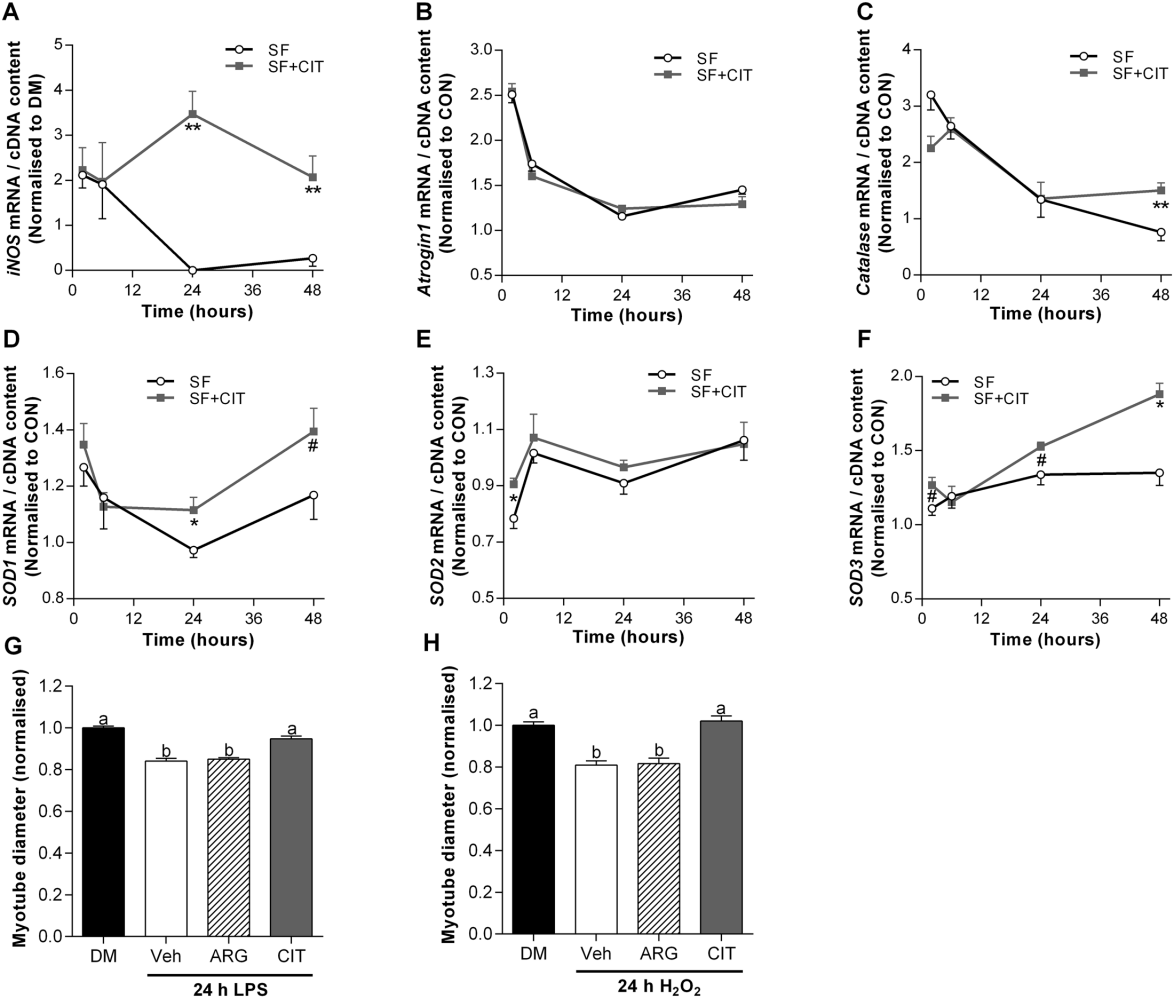
Increased *iNOS* expression is associated with increased antioxidant gene expression and L-citrulline protects muscle cells from inflammation and oxidative stress-induced myotube wasting. mRNA expression of *iNOS* (A); *atrogin-1* (B); *catalase* (C); *SOD1* (D); *SOD2* (E) and *SOD3* (F) for DM and after 24 h in serum free (SF) media treated with vehicle, 2.5 mM L-arginine (ARG) or 2.5 mM L-citrulline (CIT). Myotube diameter for cells incubated in differentiation media (DM) or: 1 μg·ml^-1^ lipopolysaccharide (LPS) for 24 h (G) or; 25 nM H_2_O_2_ for 24 h (H). For (G) and (H), cells were treated with a PBS vehicle (Veh) or 2.5 mM L-arginine or L-citrulline. Values are means ± SE, n = 4–6 per group. For (A-F) comparisons were made using a two-way ANOVA (time × treatment) with Fisher’s LSD post-hoc test. For (G) and (H), comparisons were made using a one-way ANOVA with Tukey’s post-hoc test. Different letters denote significant differences (*P*<0.05) between groups, where a>b>c. ** and * denotes a significant difference at the P<0.01 and P<0.05 level, respectively, and # denotes a trend (0.05<P<0.1) from vehicle at the specific time point.

To test the hypothesis of a temporal link between mRNA expression of *iNOS* and *SOD1-3*, *catalase* and *atrogin-1*, we incubated cells in SF media with or without L-citrulline for 2, 6, 24 and 48 h (Fig 7). Compared to CON cells, we observed a transient 100% increase in the mRNA expression of *iNOS* within the first 6 h of incubation in SF media (P<0.05). Intriguingly, *iNOS* mRNA expression was further increased after 24 h (~250% v CON, P<0.01) and 48 h (~100% v CON, P<0.05) in SF media with L-citrulline treatment, but dramatically reduced and barely detectable in vehicle treated cells (P<0.01). A concomitant improvement in antioxidant gene expression was also observed in L-citrulline treated compared to vehicle treated cells between 24 and 48 h. Compared to vehicle, *SOD1* mRNA expression was ~15% higher in L-citrulline treated cells after 24 h (P<0.05) and tended to be higher after 48 h (~19%, P = 0.09). Similarly, *SOD3* mRNA expression tended to be higher (~14%, P = 0.10) after 24 h and was 39% higher (P<0.05) after 48 h incubation in SF media treated with L-citrulline than vehicle. After 48 h, *catalase* was also ~100% higher in L-citrulline than vehicle treated cells (P<0.05). Despite the changes in *iNOS*, *SOD1*, *SOD3* and *catalase*, *atrogin 1* mRNA was not different between L-citrulline and vehicle treated cells incubated in SF at any time point. To determine whether L-citrulline could also protect muscle cells from inflammation and oxidative stress-induced myotube wasting, we induced wasting using LPS or H_2_O_2_. In both cases, L-citrulline treatment prevented myotube wasting (Fig 7G and 7H).

## Discussion

The favorable whole-body metabolic properties of L-citrulline have led to the suggestion that L-citrulline supplementation to increase muscle L-arginine concentration may represent an effective anabolic treatment [27]. In rodents, L-citrulline effectively restores muscle L-arginine stores and reduces muscle wasting in L-arginine-deficient and low-protein intake conditions [6, 11, 12]. However, it is currently unknown what effect increasing L-citrulline availability has on skeletal muscle directly. Using cultured C2C12 myotubes, we show for the first time that L-citrulline protects skeletal muscle myotubes directly from a range of cachectic stimuli. Furthermore, we show a novel role for L-citrulline in the regulation of iNOS and endogenous antioxidants in skeletal muscle cells.

### L-citrulline protects myotubes from muscle cell wasting

We observed a dose-dependent attenuation of myotube wasting, induced by growth factor and nutrient deprivation (HBS), with L-citrulline administration. Furthermore, L-citrulline protected C2C12 myotubes from multiple cachectic stimuli, including growth factor deprivation (SF), inflammation (LPS) and oxidative stress (H_2_O_2_). This is the first demonstration of a direct role for L-citrulline in the protection of skeletal muscle from wasting *in vitro*. Our observations suggest that concentrations of L-citrulline ≥1 mM are required to elicit protection from HBS-induced myotube wasting. Importantly, this concentration is above the normal baseline plasma L-citrulline concentrations of ≤40 μM in humans [5, 8, 28] and ~80 μM in rodents [29]. However, a concentration of 1 mM L-citrulline is achievable through supplementation. Ingestion of 3–10 g of L-citrulline increases plasma L-citrulline levels to 0.85–2.0 mM in humans [5, 8] and plasma L-citrulline concentrations reach ~0.8 mM 1 h after an oral gavage of 1 g·kg^-1^ L-citrulline in mice [29]. Together, these data suggest that increasing L-citrulline availability may be beneficial in muscle wasting conditions.

### The protective effect of L-citrulline treatment is not analogous with L-arginine treatment

We have shown that L-arginine protects C2C12 skeletal muscle myotubes from wasting [14] and therefore used isomolar concentrations of L-arginine as a positive control in our *in vitro* models of muscle wasting. While L-arginine attenuated HBS-induced myotube wasting, confirming our previous study [14], no effect of L-arginine was observed in the SF, LPS or H_2_O_2_ models of myotube wasting. Moreover, equimolar concentrations of the non-essential amino acid L-alanine did not provide protection from myotube wasting, indicating an amino acid specific effect of L-citrulline. These observations contrast with those in smooth muscle cells where L-citrulline is rapidly recycled to L-arginine by the enzymes ASS1 and ASL and the effects of exogenous L-citrulline are analogous to those of L-arginine [16]. To further investigate whether L-citrulline can efficiently restore L-arginine availability in skeletal muscle cells, we incubated myotubes in L-arginine free SF media. We confirmed that L-arginine deprivation exacerbated SF-induced myotube wasting [14], and that this was not restored by L-citrulline treatment (Fig 3). The failure of L-citrulline to protect cells from wasting during L-arginine deficient conditions may be attributed to an inability to produce NO or a reduced capacity to synthesize new proteins. Regardless of the exact mechanism, our findings reveal that exogenous L-citrulline cannot efficiently restore L-arginine availability in cultured skeletal muscle cells. Thus, in cultured skeletal muscle cells L-arginine can be considered an essential amino acid and is an important substrate for the synthesis of new proteins, and is the primary substrate for the production of NO by NOS. Therefore, a deficiency in L-arginine limits NO production and the rate of protein synthesis and cell growth.

### The protective effect of L-citrulline is mediated through improvements in protein synthesis

In SF media, we observed a transient increase in the mRNA expression of the key muscle specific E3 ubiquitin ligase *atrogin-1*, which rapidly returned to basal levels between 6 and 24 h. In contrast, we demonstrated a 26% reduction in protein synthesis after prolonged (48 h) incubation in SF media. These findings are reminiscent of those from bed rest and immobilization studies in humans where, after a transient upregulation in muscle breakdown, reductions in protein synthesis are primarily responsible for the loss of muscle tissue [30–32]. Interestingly, L-citrulline completely prevented the SF-induced reduction in protein synthesis (Fig 2A). Furthermore, impeding the synthesis of new proteins by depriving cells of the essential amino acid leucine, prevents the positive effects of L-citrulline (Fig 3). This suggests that L-citrulline’s protective effects are not mediated through a reduction in protein breakdown. It is well known that cells require essential amino acids such as leucine for de novo protein synthesis and removing any single essential amino acid will blunt protein synthesis. While the failure of L-citrulline to preserve myotube diameter during leucine deprivation does not unveil the specific protective mechanism of L-citrulline, the results further highlight that the citrulline-induced increase in protein synthesis (Fig 2A) is necessary for its protective effect. Similarly, compared to L-alanine, protein synthesis was higher in L-citrulline treated cells after both 1 and 4 h incubation in HBS, which was associated with an increased phosphorylation of mTOR and 4EBP1 in L-citrulline treated cells after 1 h incubation in HBS. However, despite a higher phosphorylation status of mTOR and 4EBP1, mTORC1 activation does not appear critical to the protective effect of L-citrulline since the mTORC1 inhibitor rapamycin did not prevent the protective effect of L-citrulline.

### L-citrulline increases the mRNA expression of *iNOS*

There are three NOS isoforms expressed in skeletal muscle; two constitutively active isoforms, endothelial NOS (eNOS) and neuronal NOS (nNOS), and one inducible isoform (iNOS). We observed that L-citrulline administration to C2C12 myotubes increased the mRNA expression of *iNOS*, without altering the expression of *eNOS* and *nNOS* (Fig 4), while protein levels of iNOS remain below detection levels, which is in line with previous observations where an inflammatory insult was required for the detection of iNOS protein [22]. The traditional view that iNOS is a pro-cachectic factor that contributes to skeletal muscle wasting [33, 34] is based on the observation that whole-body ablation of the iNOS gene inhibits the cachectic response in skeletal muscle to an endotoxin challenge in mice [35, 36]. However, these effects are likely attributable to the established role of iNOS in inflammatory signaling in macrophages and the role of skeletal muscle iNOS cannot be determined from these mouse models. In contrast to macrophage iNOS, evidence is mounting that iNOS expressed in skeletal and cardiac muscle plays an important role in muscle regeneration [19] and in the endogenous anti-oxidant defense system that protects cells from cachectic stimuli [22, 37–40].

Our use of cultured skeletal muscle cells obviates the potential influence of macrophages, and is a useful model to study the muscle-specific responses to changes in L-citrulline availability. In skeletal muscle, Yu et al. [22] observed enhanced iNOS expression in oxidative compared to glycolytic muscles and linked this characteristic to their inherent resistance to chronic heart failure-induced cachexia. Furthermore, the potent cytoprotective effects of the drug Sildenafil are mediated in part through enhanced iNOS expression [38]. To our knowledge, this is the first study to report a nutrient-induced upregulation of iNOS mRNA expression in skeletal muscle cells, and has potential implications for numerous muscle wasting conditions. It is important to note, however, that iNOS expression in inflammatory cells such as macrophages plays a pro-cachectic role in a number of muscle wasting conditions such as muscular dystrophy [41] and further work is required to determine the effect of citrulline supplementation on iNOS protein expression and activity during inflammatory muscle wasting conditions.

### *iNOS* activity is necessary for L-citrulline’s protective effect

To determine how meaningful the observed increase in iNOS mRNA was to the protective effect of L-citrulline, we undertook a number of experiments. Firstly, using the general NOS inhibitor L-NAME and the iNOS specific inhibitor aminoguanidine, we determined that iNOS activity is critical for the protective effects of L-citrulline in both HBS (Fig 4) and SF (Fig 5) models of myotube wasting. Secondly, we treated cells in SF media with 0.2 mM of the nitric oxide donor SNP (Fig 6). As with L-citrulline treatment, SNP preserved myotube diameter in cells incubated in SF media. This is consistent with previous work showing that provision of exogenous NO to cultured muscle cells using the NO-donor diethylenetriamine NO (DETA-NO), enhanced *iNOS* expression and completely prevented the induction of atrogin-1 by TNF-α [22]. Likewise, injection of the endogenous NO-donor S-nitrosoglutathione (GSNO) induced *iNOS* expression and prevented the LPS-stimulated increase in *atrogin-1* mRNA in mouse plantaris muscles [22]. Together, these results demonstrate that L-citrulline protects skeletal muscle cells in a NO-dependent manner.

### Increased *iNOS* expression is associated with increased antioxidant gene expression

In skeletal muscle, iNOS promotes the induction of antioxidant genes, specifically the super oxide dismutase isoforms (SOD 1–3) and catalase, and reduces the expression of the muscle specific ubiquitin E3 ligase atrogin-1 [22]. Increased production of reactive oxygen species (ROS) is a hallmark of many muscle wasting conditions [42]. ROS can damage lipids, DNA and proteins and can activate catabolic processes [43] and inhibit protein synthesis [44] in skeletal muscle. The SOD family of proteins plays a key role in the endogenous antioxidant defense in skeletal muscle. For example, adult SOD1^-/-^ mice experience greater levels of oxidative damage [45] and an accelerated age-related decline in muscle mass and function [46]. We hypothesized a temporal link between the mRNA expression of *iNOS*, *atrogin-1* and antioxidant genes during SF-induced muscle cell wasting. Compared to CON cells, we observed a transient increase in the mRNA expression of *iNOS* within the first 6 h of incubation in SF media. Intriguingly, by 24 h, *iNOS* mRNA expression was further increased in L-citrulline treated cells, but dramatically reduced and barely detectable in vehicle treated cells. A concomitant improvement in antioxidant gene expression was also observed in L-citrulline treated compared to vehicle treated cells between 24 and 48 h. *SOD1* and *SOD3* were significantly higher or tended to be higher in L-citrulline treated cells after 24 and 48 h, and catalase was significantly higher after 48 h. Despite the associated changes in antioxidant gene expression, increased *iNOS* mRNA expression was not associated with changes in *atrogin-1* mRNA. Together with the higher mRNA expression of *iNOS*, the higher mRNA expression of *SOD* and *catalase* may indicate an enhanced antioxidant defense system that could protect cells from oxidative stress. Our current understanding regarding the role of citrulline and iNOS in the modulation of skeletal muscle antioxidant defense mechanisms is incomplete and warrants further investigation. The antioxidant properties of L-citrulline were first described in the leaves of drought resistant wild watermelon plants [47]. Furthermore, markers of improved redox status have been shown with aged rates [48] and in patients with endothelial dysfunction [49]. In addition, we showed that supplemental L-citrulline reduces myotube wasting induced by the free radical hydrogen peroxide and the endotoxin lipopolysaccharide (LPS, Fig 1). While the increased expression of antioxidant genes suggests a citrulline-induced activation of the antioxidant program, more detailed work, including determining antioxidant content, redox status and enzyme activity, is required to make a definitive statement about the causative effect of the observed changes in antioxidant enzyme mRNA.

## Conclusion

In conclusion, L-citrulline preserves rates of protein synthesis and protects myotubes from wasting in a different manner than L-arginine. We demonstrate a novel direct protective effect of L-citrulline on protein metabolism and skeletal muscle cell size that is not mediated by signaling through the mechanistic Target of Rapamycin. Instead, we show these effects are mediated through the inducible NOS (iNOS) isoform. This is the first study to describe a nutritional modulator of *iNOS* mRNA expression by skeletal muscle cells and could have important implications for the treatment of muscle wasting conditions.

## Supporting Information

S1 Fig Cell viability. Brightfield images of unstained myotubes (CON) and images of compromised cells stained with trypan blue (red) in DMEM and 2% horse serum (DM) or serum free DMEM with L-alanine (SF + ALA) or L-citrulline (SF + CIT). While a few trypan blue positive myotubes could be found in both DM (DM^+^) and serum free (SF^+^) conditions, when four images were taken per well from pre-defined locations across three separate wells (as described in the methods), no trypan blue staining was observed in any of the three treatment groups (DM, SF + ALA and SF + CIT). Given the number of fibres per image are 10–15, this represents a percentage of trypan blue positive myotubes of <0.5–1%.
(DOCX)
