# Supplementary figures and images for: L-Citrulline Protects Skeletal Muscle Cells from Cachectic Stimuli through an iNOS-Dependent Mechanism
Source: PLoS One. 2015 Oct 29;10(10):e0141572. doi: 10.1371/journal.pone.0141572 (PMC4625972; doi:10.1371/journal.pone.0141572)

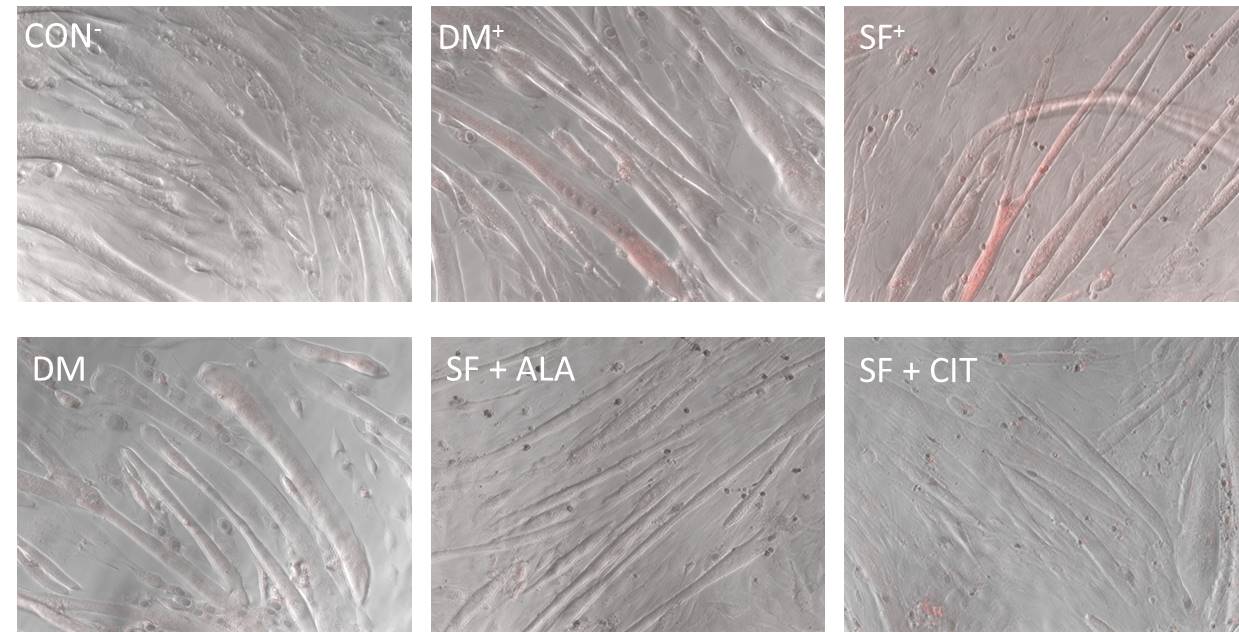


**S1 Fig. Cell viability.**

Supplement: S1 Fig — Brightfield images of unstained myotubes (CON) and images of compromised cells stained with trypan blue (red) in DMEM and 2% horse serum (DM) or serum free DMEM with L-alanine (SF + ALA) or L-citrulline (SF + CIT). While a few trypan blue positive myotubes could be found in both DM (DM+) and serum free (SF+) conditions, when four images were taken per well from pre-defined locations across three separate wells (as described in the methods), no trypan blue staining was observed in any of the three treatment groups (DM, SF + ALA and SF + CIT). Given the number of fibres per image are 10–15, this represents a percentage of trypan blue positive myotubes of <0.5–1%. (DOCX) [file pone.0141572.s001.docx]
